# A detailed view on 1,8-cineol biosynthesis by *Streptomyces clavuligerus*

**DOI:** 10.3762/bjoc.12.225

**Published:** 2016-11-04

**Authors:** Jan Rinkel, Patrick Rabe, Laura zur Horst, Jeroen S Dickschat

**Affiliations:** 1 Kekulé-Institute of Organic Chemistry and Biochemistry, University of Bonn, Gerhard-Domagk-Straße 1, 53121 Bonn, Germany

**Keywords:** biosynthesis, enzyme mechanisms, isotopic labelling, stereochemistry, terpenes

## Abstract

The stereochemical course of the cyclisation reaction catalysed by the bacterial 1,8-cineol synthase from *Streptomyces clavuligerus* was investigated using stereospecifically deuterated substrates. In contrast to the well investigated plant enzyme from *Salvia officinalis*, the reaction proceeds via (*S*)-linalyl diphosphate and the (*S*)-terpinyl cation, while the final cyclisation reaction is in both cases a *syn* addition, as could be shown by incubation of (2-^13^C)geranyl diphosphate in deuterium oxide.

## Introduction

**Figure 1:**
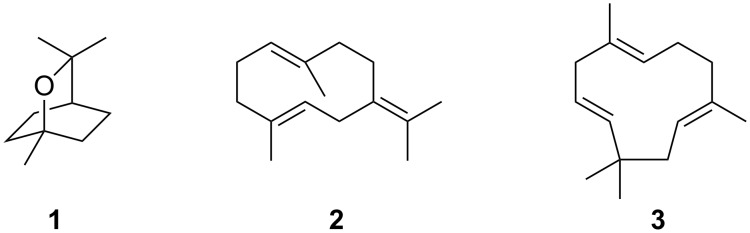
Selection of achiral terpenes.

Among all classes of natural products the climax of structural diversity and complexity is reached within the largest, the terpenoids. An estimated number of 75,000 different compounds are known from all kinds of organisms including plants [1], bacteria [2–5], fungi [6] and, as recently shown, even social amoebae [7]. These molecules are all made from only a handful of linear and achiral precursors such as geranyl diphosphate (GPP, monoterpenes), farnesyl diphosphate (FPP, sesquiterpenes) and geranylgeranyl diphosphate (GGPP, diterpenes). Terpene cyclases (type I) contain a trinuclear (Mg^2+^)_3_ cluster in their active site that is stabilised by binding to several highly conserved motifs including the aspartate-rich motif (DDXXD) and the NSE triad (ND(L,I,V)XSXXXE, modified in plants to a DTE triad: DD(L,I,V)XTXXXE) [8]. Their substrates bind with the diphosphate portion to the (Mg^2+^)_3_ cluster and via hydrogen bridges to a highly conserved arginine (diphosphate sensor) and a RY dimer [9]. The substrate is ionised by diphosphate abstraction and the resulting allyl cation undergoes a domino reaction via a series of cationic intermediates and a final deprotonation or attack of water to yield a terpene hydrocarbon or alcohol. This reaction cascade proceeds in a hydrophobic cavity from which water is excluded to enable carbocation chemistry in an aqueous environment. Furthermore, the hydrophobic cavity provides a template that arranges the substrate in a certain conformation to determine the formation of a specific product. Single residues such as phenylalanines are involved in the stabilisation of cationic intermediates, e.g., by cation–π interactions [8–10]. The overall process usually generates an enantiomerically pure (poly)cyclic terpene with several stereogenic centres. A large variety of carbon skeletons is accessible, e g., more than 120 skeletons each representing various stereoisomers and constitutional isomers with different positioning of olefinic double bonds or alcohol functions are known just for sesquiterpenes [11]. The structural diversity of terpenoids can be further increased by the action of tailoring enzymes such as cytochrome P450 monooxygenases and acyl transferases [12–13]. Very few cases are known in which terpene cyclases generate an achiral product as exemplified by the monoterpene 1,8-cineol (eucalyptol, **1**) and the sesquiterpenes germacrene B (**2**) and α-humulene (**3**) (Figure 1).

**Scheme 1:**
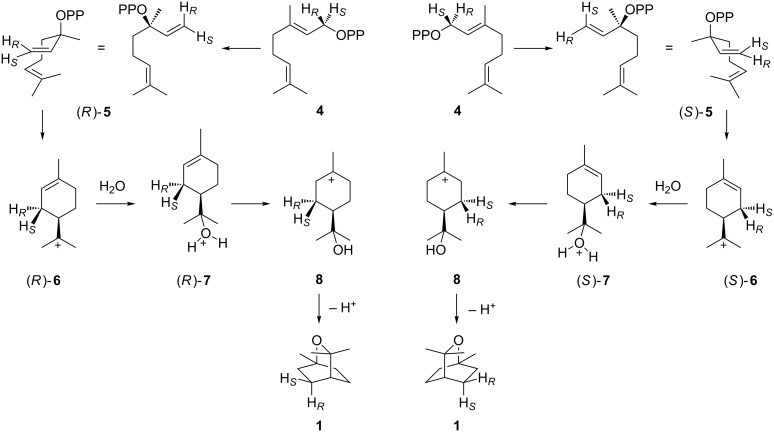
Cyclisation of GPP to **1** via the (*R*)-terpinyl cation ((*R*)-**6**, left) or the (*S*)-terpinyl cation ((*S*)-**6**, right).

A direct 1,6-cyclisation of the monoterpene precursor GPP to **1** is prevented by the topological constraints associated with the (2*E*) geometry which necessitates the isomerisation of GPP (**4**) to linalyl diphosphate (LPP, **5**) followed by an *anti*,*endo*-S_N_’-cyclisation [14], but the stereochemical course of this reaction is not readily clear and may proceed via either enantiomer of the α-terpinyl cation (**6**, Scheme 1). Isotopic labelling experiments currently experience a revival [15] and are a very powerful method to follow the enzyme mechanisms of terpene cyclases [16–24] including the stereochemical courses of the cyclisation reactions (reviewed in [25]). While the stereochemical course of the GPP cyclisation to **1** has been investigated for the 1,8-cineol synthase from *Salvia officinalis* [26–27], it is unknown for the bacterial enzyme that was recently reported from *Streptomyces clavuligerus* [28]. Here we describe isotopic labelling experiments that gave insights into the cyclisation mechanism of the bacterial 1,8-cineol synthase.

## Results

### The absolute configuration of the intermediate terpinyl cation

**Figure 2:**
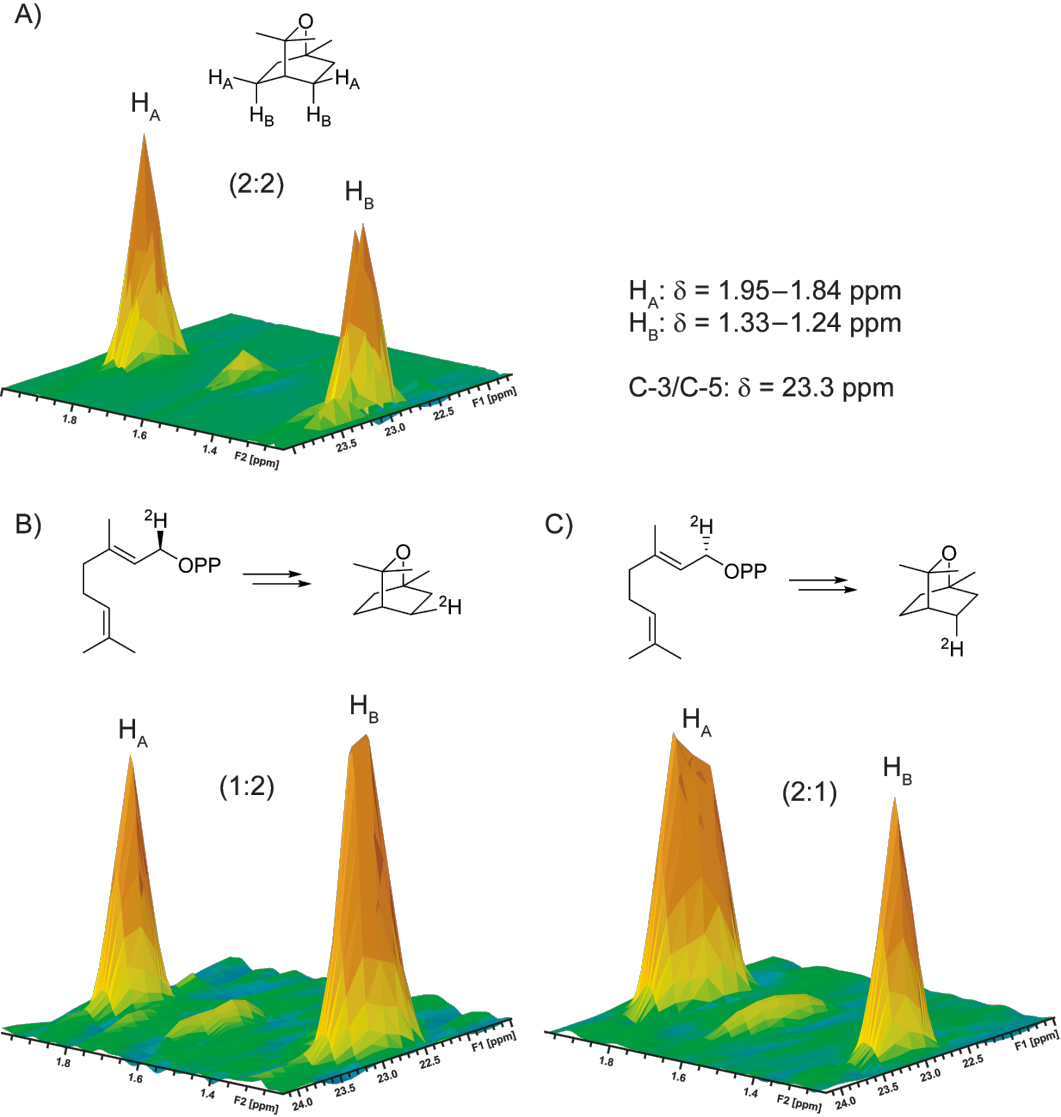
Partial HSQC spectra showing the region of crosspeaks for H_A_ and H_B_ connected to C-3 and C-5 of A) unlabelled **1**, B) (^2^H)-**1** obtained by enzymatic conversion of (*R*)-(1-^2^H)GPP, and C) (^2^H)-**1** obtained by enzymatic conversion of (*S*)-(1-^2^H)GPP. The indicated chemical shift data are for unlabelled **1** (for full data cf. Table S1, Supporting Information File 1).

While the two possible cyclisation pathways via (*R*)- and (*S*)-**6** to **1** cannot be distinguished with unlabelled GPP, its two enantiotopic protons at C-1 (indicated by H*_R_* for the *pro*-*R* hydrogen and H*_S_* for the *pro*-*S* hydrogen) end up in diastereotopic positions of **1**. Thus, a labelling experiment using the deuterated substrates (*R*)-(1-^2^H)GPP (H*_R_* = ^2^H, H*_S_* = H) and (*S*)-(1-^2^H)GPP (H*_R_* = H, H*_S_* = ^2^H) can give insights whether the cyclisation proceeds via (*R*)- or (*S*)-**6**, by determination in which of the distinguishable diastereotopic positions the label ends up. The synthesis of the two enantiomers of (1-^2^H)GPP (Scheme S1, Supporting Information File 1) was performed by Alpine borane reduction [29] (both enantiomers of this reagent are commercially available) of (1-^2^H)geranial to (*R*)- and (*S*)-(1-^2^H)geraniol that were obtained with high enantiomeric excess (>95% ee) as determined by Mosher ester analysis (Figure S1, Supporting Information File 1). The alcohols were subsequently converted into the corresponding diphosphates using triethylammonium phosphate in trichloroacetonitrile [30–31]. The gene encoding the 1,8-cineol synthase [28] was cloned into the yeast-to-*Escherichia coli* shuttle vector pYE-Express by homologous recombination in yeast [32], followed by expression in *E. coli* BL21. The protein was purified by Ni^2+^-NTA affinity chromatography and used to convert both (*R*)- and (*S*)-(1-^2^H)GPP into (^2^H)-**1** (in agreement with the findings described in reference [28], **1** is the only product from unlabelled GPP as was shown by GC–MS, Figure S2, Supporting Information File 1). The obtained products were analysed by HSQC spectroscopy (Figure 2). While for unlabelled **1** a 2:2 signal intensity is observed for the crosspeaks representing the two pairs of enantiotopic hydrogens H_A_ and H_B_ connected to carbons C-3 and C-5, the sample obtained from (*R*)-(1-^2^H)GPP gave a 1:2 ratio of signal intensities (i.e., H_A_ = ^2^H), while the sample from (*S*)-(1-^2^H)GPP resulted in ratio of 2:1 by peak integration (i.e., H_B_ = ^2^H), indicating the cyclisation via (*S*)-LPP ((*S*)-**5**) and the (*S*)-terpinyl cation ((*S*)-**6**) (Scheme 1, right).

### *Syn* versus *anti* addition in the final ring closure

**Figure 3:**
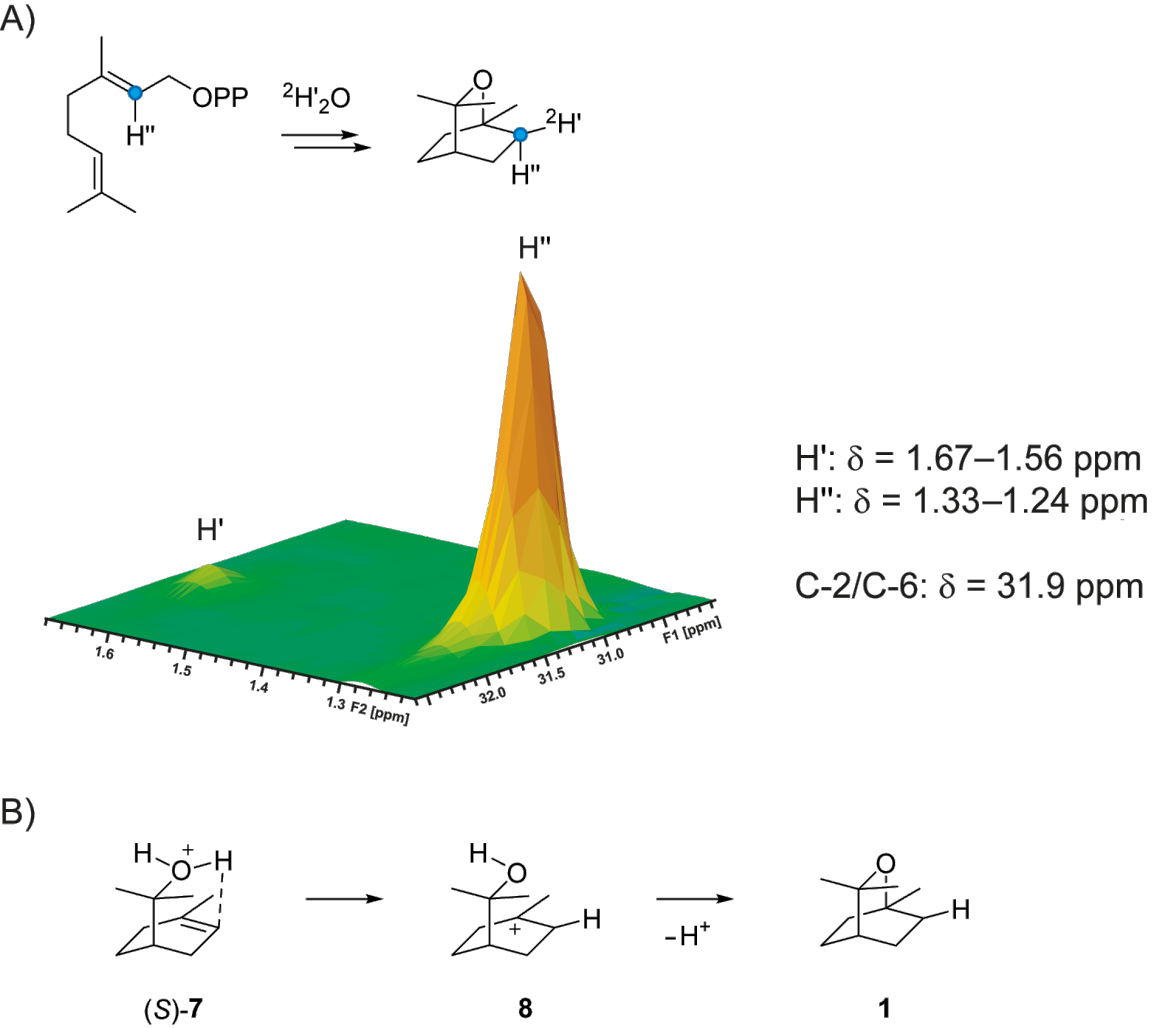
A) Partial HSQC spectrum showing the region of crosspeaks of C-2 with its directly connected hydrogens H’ and H’’ for compound (2-^13^C,2-^2^H)-**1** obtained by enzymatic conversion of (2-^13^C)GPP in deuterium oxide. Deuterium was specifically incorporated into H’ position. The indicated chemical shift data are for unlabelled **1** (for full data cf. Table S1, Supporting Information File 1). Blue circles point to ^13^C-labelled carbons. B) Intramolecular proton transfer from the protonated hydroxy function in (*S*)-**7** to C-2.

The final cyclisation step from (*S*)-**7** via **8** to **1** can in principle proceed either via a *syn* or an *anti* addition to the olefinic double bond, requiring a protonation of the original C-2 of GPP. To distinguish between these alternatives (2-^13^C)GPP was synthesised from sulcatone (Scheme S2, Supporting Information File 1) and converted by the 1,8-cineol synthase in deuterium oxide. The obtained product was analysed by HSQC spectroscopy (Figure 3A), showing that deuterium is taken up into the *exo* position at the ^13^C-labelled C-2 of **1** (indicated by H’), while the *endo* position (H’’) is occupied by the proton from the substrate, resulting in a strong crosspeak [22]. Furthermore, deuterium incorporation at C-2 was indicated by a strongly enhanced triplet in the ^13^C NMR spectrum due to ^13^C-^2^H-spin coupling (Figure S4, Supporting Information File 1) [11,20,23]. This finding is in agreement with a *syn* addition to the olefinic double bond of (*S*)-**7** in the final cyclisation step. It is possible that the proton is directly transfered from the protonated hydroxy function in (*S*)-**7** to C-2 (Figure 3B). Alternatively, a deprotonation of (*S*)-**7** to the hypothetical neutral intermediate α-terpineol followed by reprotonation at C-2 from the *Si* face can be assumed, but these two alternatives cannot be distinguished based on the labelling experiments described here.

## Discussion

**Scheme 2:**
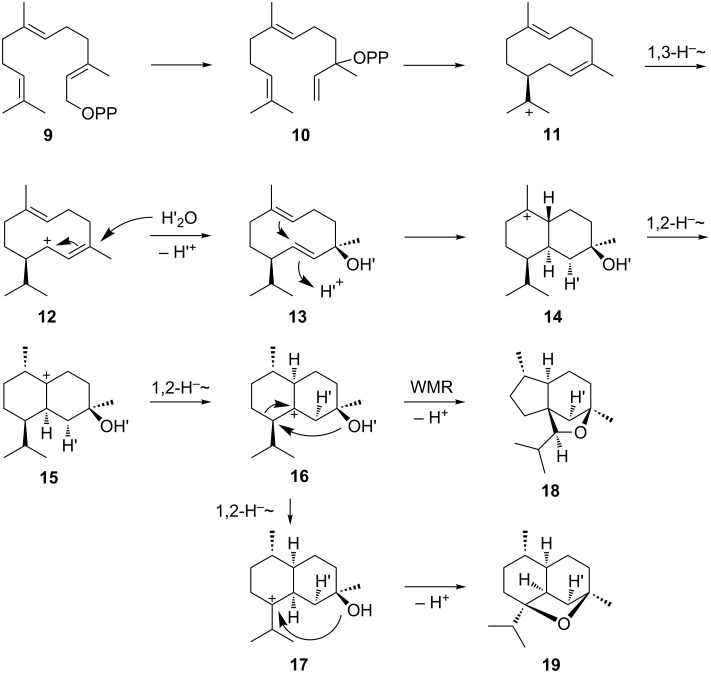
Mechanism for the cyclisation of FPP to corvol ethers A (**19**) and B (**18**). WMR: Wagner-Meerwein rearrangement.

Plant and bacterial terpene cyclases show important structural differences [33]. While plant monoterpene synthases are composed of α and β domains and exhibit a quarternary α_2_β_2_ structure [34–35], bacterial mono- and sesquiterpene cyclases are monodomain enzymes (α) [9–10,36]. Accordingly, also the 1,8-cineol synthases from *Salvia officinalis* and from *Streptomyces clavuligerus* are not related and have evolved independently. While the plant enzyme was shown to convert GPP via (*R*)-LPP ((*R*)-**5**) and the (*R*)-terpinyl cation ((*R*)-**6**) into **1** [27], the experiments described here revealed a different course for the bacterial enzyme via (*S*)-**6**. This finding is particularly interesting, because it reflects the frequent observation that the (chiral) products of bacterial terpene cyclases represent the opposite enantiomers as usually generated by plant enzymes [24,37–38]. Both enzymes from *Salvia officinalis* and from *Streptomyces clavuligerus* share the *syn* addition in the final cyclisation step which can be rationalised by a direct intramolecular proton transfer, circumventing the need of a low-energy neutral intermediate such as α-terpineol. However, in case of the sesquiterpene ethers corvol ethers A (**19**) and B (**18**) a reprotonation step was shown to proceed from the opposite face than the preceeding attack of water, thus excluding a direct proton transfer from oxygen to the neighbouring carbon (reactions from **12** to **14** in Scheme 2). Conclusively, reprotonation of the neutral intermediate **13** is possible in this case [21].

## Experimental

### Cloning and homologous recombination

Cells of *Streptomyces clavuligerus* ATCC 27064 were obtained from the Deutsche Sammlung von Mikroorganismen und Zellkulturen (DSMZ, Braunschweig, Germany). Cultivation was done in liquid 65 Gym medium (4 g yeast extract, 4 g glucose, 10 g malt extract, 1 L water, pH 7.2) and isolation of genomic DNA was performed using a standard protocol. The gene WP_003952918 encoding the 1,8-cineol synthase was amplified using forward primer (ATGCCCGCCGGCCACGAAGA) and reversed primer (TCACCAAGGGGTGGTGGCCC). The isolated PCR product was elongated for homologous recombination with pYE-Express in yeast with a second set of primers (GGCAGCCATATGGCTAGCATGACTGGTGGAATGCCCGCCGGCCACGAAGA and TCTCAGTGGTGGTGGTGGTGGTGCTCGAGTTCACCAAGGGGTGGTGG CCC). This elongated product was transformed in *Saccharomyces cerevisiae* FY834 together with linearised vector pYE-Express [32] (EcoRI and HindIII digestion) using the LiOAc/SS carrier DNA protocol [39]. Transformed cells were plated on SM-URA medium (20 g glucose, 1.7 g yeast nitrogen base, 5 g ammonium sulphate, 0.77 g nutritional supplement minus uracil, 24 g agar, 1 L water) and grown for 3 days at 28 °C. Plasmids were isolated using the kit Zymoprep Yeast Plasmid Miniprep II (Zymo Research, Irvine, USA), shuttled in *E. coli* BL21 by electroporation and confirmed by sequencing.

### Incubation experiments with (1*R*)- and (1*S*)-(1-^2^H)GPP

A preculture of *E. coli* BL21 cells carrying the plasmid pYE_WP003952918 in 2YT medium (16 g trypton, 10 g yeast extract, 5 g NaCl, 1 L water, pH 7.2) was grown overnight to inoculate a 2YT main culture (2 L). The cultures were shaken at 160 rpm, 37 °C until OD_600_ = 0.4 was reached. Prior to induction with IPTG (0.4 mM), the cultures were cooled down to 18 °C. After incubation overnight (160 rpm, 18 °C), cells were harvested by centrifugation (5000*g*, 4 °C, 45 min) and resuspended in binding buffer (20 mL; 20 mM Na_2_HPO_4_, 0.5 mM NaCl, 20 mM imidazole, 1 mM MgCl_2_, pH 7.0). The cells were crushed by ultra-sonification (6 × 1 min, 4 °C) and the cell debris pellet was separated by centrifugation (10 min, 15500*g*, 4 °C). The soluble fraction was loaded onto a Ni^2+^-NTA affinity column (Novagen) and treated with binding buffer (2 × 10 mL). The target protein was then eluted with elution buffer (2 × 10 mL; 20 mM Na_2_HPO_4_, 0.5 M NaCl, 0.5 M imidazole, 1 mM MgCl_2_, pH 7.0) and used directly for incubations with 5 mg of (1*R*)- and (1*S*)-(1-^2^H)GPP, solved in incubation buffer (50 mM Tris/HCl, 10 mM MgCl_2_, 20% v/v glycerol, pH 8.2) to reach a final substrate concentration of 0.2 mg/mL. The enzyme reaction was incubated for 2 h at 28 h, overlaid with 400 μL (^2^H_6_)benzene and further incubated overnight. The organic phase was separated, dried over MgSO_4_ and directly analysed by GC–MS and NMR.

### Incubation experiment with (2-^13^C)GPP

Enzyme purification starting from an *E. coli* expression culture (0.5 L) was performed as described above. The last washing fraction was substituted with ^2^H_2_O-based binding buffer and elution was done with ^2^H_2_O-based elution buffer. The first elution fraction was incubated with (2-^13^C)GPP (0.8 mg) for 16 h at 28 °C. The enzyme reaction was extracted with (^2^H_6_)benzene (0.6 mL), dried with MgSO_4_ and the extract was analysed directly by GC–MS and NMR.

### NMR spectroscopy

To record NMR spectra, instruments AV Avance DMX-500 (500 MHz), DPX-400 (400 MHz) and AV III HD Cryo (700 MHz) from Bruker were used. Solvent signals were used to reference the spectra (^1^H NMR, residual proton signals: (^2^H_6_)benzene δ = 7.16; ^13^C NMR: (^2^H_6_)benzene δ = 128.06) [40].

### GC–MS analysis

An Agilent 7890B gas chromatograph equipped with a HP5-MS silica column (30 m, 0.25 mm inner diameter, 0.50 μm film) connected to an Agilent 5977A inert mass selective detector was used to acquire GC–MS data. Instrumental settings were: (1) inlet pressure: 77.1 kPa, He: 23.3 mL/min, (2) transfer line: 250 °C, (3) electron energy: 70 eV. The GC was set to 50 °C starting temperature for 5 min, then increasing with 5 °C per minute to 320 °C and holding this temperature for another 5 min. The injection volume was 2 μL and the inlet was operating in split mode (10:1, 60 s valve time). Helium was used as the carrier gas at 1 mL/min. Retention indices were determined against a homologous series of *n*-alkanes (C_8_–C_40_).

### Synthesis of (2-^13^C)geranyl diphosphate

(2-^13^C)Geraniol was synthesised as reported previously [41]. The synthetic (2-^13^C)geraniol (16 mg, 0.072 mmol, 1.0 equiv) was dissolved in dry THF (0.3 mL) and PBr_3_ (8.1 mg, 0.029 mmol, 0.4 equiv) was added at 0 °C. The solution was stirred for 45 min at room temperature. The reaction mixture was hydrolyzed by addition of ice cold water and extracted three times with pentane. The combined organic layers were dried over MgSO_4_ and the solvent was removed under reduced pressure. The crude product was used for phosphorylation.

In a second flask, to a solution of (*n*-Bu_4_)_3_HP_2_O_7_ (97 mg, 0.11 mmol, 1.5 equiv) in dry CH_3_CN (1.0 mL) the crude product of the allyl bromide (1.0 equiv) was added and the reaction mixture was stirred for 2 h at room temperature and then concentrated under reduced pressure. The colorless oil was loaded onto an ion exchange column (DOWEX 50W-X8, NH_4_^+^ form). Elution of the product was performed by addition of two column volumes of ion exchange buffer (0.03 M NH_4_HCO_3_ in 2% iPrOH/H_2_O). Freeze drying yielded the product as a white solid (14.1 mg, 0.04 mmol, 55%).

^1^H NMR (500 MHz, H_2_O) δ 5.37 (dt, ^1^*J*_(C,H)_ = 156.7 Hz, ^3^*J*_(H,H)_ = 6.9 Hz, 1H, CH), 5.16–5.11 (m, 1H, 1 × CH), 4.44–4.38 (m, 2H, 1 × CH_2_), 2.12–2.06 (m, 2H, 1 × CH_2_), 2.06–2.00 (m, 2H, 1 × CH_2_), 1.65 (d, ^3^*J*_(C,H)_ = 5.2 Hz, 3H, 1 × CH_3_), 1.62 (s, 3H, 1 × CH_3_), 1.56 (s, 3H, 1 × CH_3_) ppm; ^13^C NMR (125 MHz, H_2_O) δ 141.9 (d, ^1^*J*_(C,C)_ = 72.7 Hz, 1 × C_q_), 133.7 (1 × C_q_), 124.1 (1 × CH), 119.6 (d, ^3^*J*_(P,C)_ = 8.2 Hz, 1 × ^13^CH), 38.8 (d, ^2^*J*_(C,C)_ = 2.7 Hz, 1 × CH_2_), 25.6 (d, ^3^*J*_(C,C)_ = 2.9 Hz, 1 × CH_2_), 24.8 (1 × CH_3_), 16.9 (1 × CH_3_), 15.6 (d, ^2^*J*_(C,C)_ = 1.2 Hz, 1 × CH_3_) ppm; ^31^P NMR (202 MHz, H_2_O) δ −10.0 (m, 1 × P), −10.6 (m, 1 × P) ppm.

## Supporting Information

Synthesis schemes, Mosher ester analysis of (*R*)- and (*S*)-(1-^2^H)GPP, gas chromatogram of the enzyme product of 1,8-cineol synthase, ^13^C NMR of the enzyme product from (2-^13^C)GPP in deuterium oxide buffer, and full NMR data of **1**.

File 1 Additional material.
