# Supplementary material for: A detailed view on 1,8-cineol biosynthesis by Streptomyces clavuligerus
Source: Beilstein J Org Chem. 2016 Nov 4;12:2317–24. doi: 10.3762/bjoc.12.225 (PMC5238540; doi:10.3762/bjoc.12.225)
Supplement: File 1 — Additional material. [file Beilstein_J_Org_Chem-12-2317-s001.pdf]

Supporting Information

for

A detailed view on 1,8-cineol biosynthesis by Streptomyces clavuligerus

Jan Rinkel, Patrick Rabe, Laura zur Horst and Jeroen S. Dickschat*

Address: Kekulé-Institute of Organic Chemistry and Biochemistry, University of Bonn, Gerhard-Domagk-Straße 1, 53121 Bonn, Germany

Email: Jeroen S. Dickschat - dickschat@uni-bonn.de

* Corresponding author

Additional material

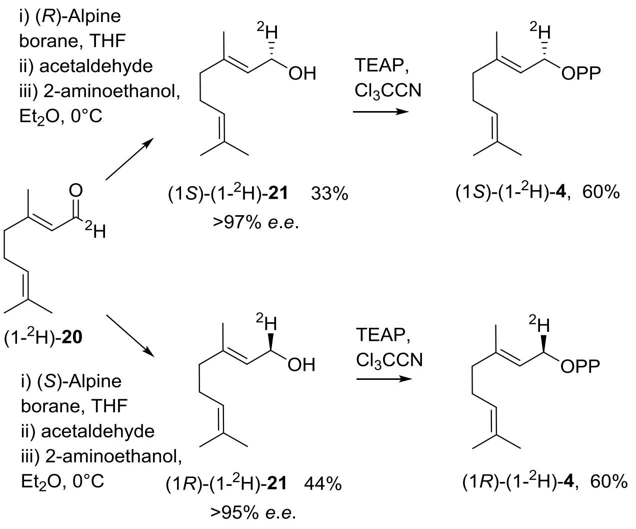

Scheme S1: Synthesis of (1*R*)- and (1*S*)-(1-^{2}H)GPP starting from (^{2}H)geranial via known procedures (TEAP: triethylammonium phosphate) [1].

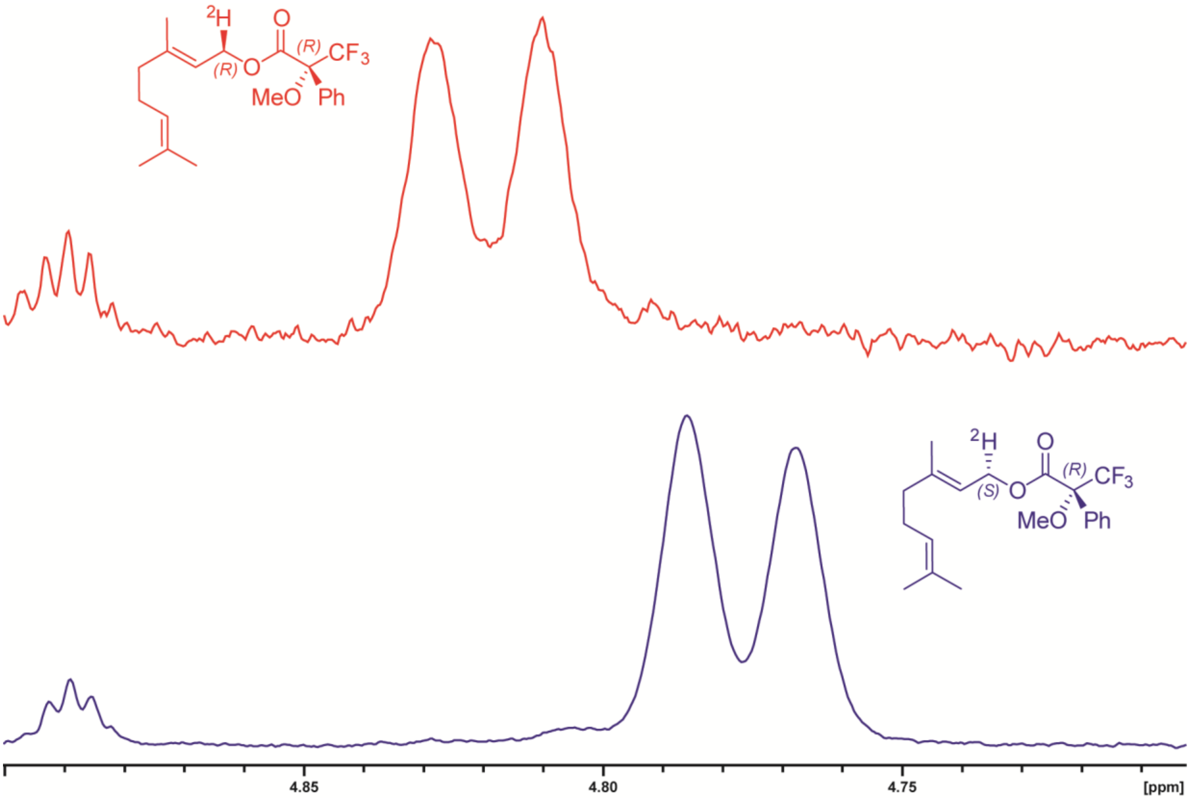

Figure S1: ^{1}H NMR of corresponding Mosher esters to A) (1*R*)-(1-^{2}H)-**4** and B) (1*S*)-(1-^{2}H)-**4** synthesised by using (*S*)-(+)-MTPA-Cl on an analytical scale [2]. The signals of C-1 proton at 4.82 and 4.78 ppm for the diastereomers indicate the enantiomeric purity of the samples. Enantiomeric excesses of the starting materials were determined by peak integration to be >95% ee for the (1*R*) sample and >97% ee for the (1*S*) sample.

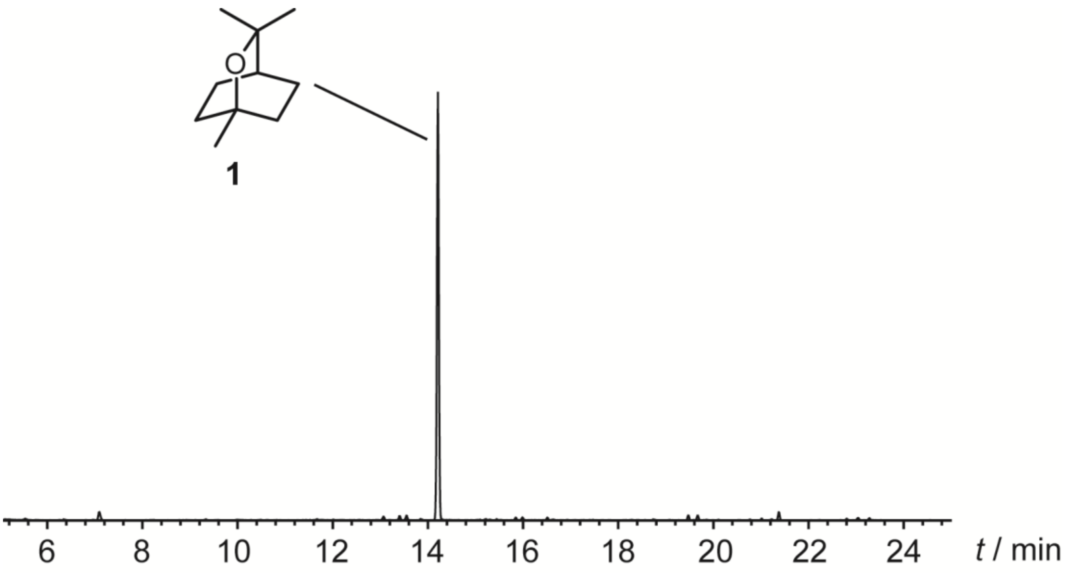

Figure S2: GC–MS (TIC) chromatogram of a hexane extract of GPP incubation with purified 1,8-cineol synthase WP_003952918 showing main product 1,8-cineol (**1**) with a retention index of *I* = 1033 (HP-5, lit.: 1033 [3]).

Table S1: NMR data of 1,8-cineol (**1**) recorded in ($^2H_6$)benzene.

C[a]	^{13}C (δ)[b]	^1H (δ, m, int)[c]
1	69.5 (C_q)	–
2,6	31.9 (CH_2)	1.67-1.56 (m, 2H)<br>1.33-1.24 (m, 2H)
3,5	23.3 (CH_2)	1.95-1.84 (m, 2H)<br>1.33-1.24 (m, 2H)
4	33.2 (CH)	1.16-1.13 (m, 1H)
7	27.9 (CH_3)	1.09 (s, 3H)
8	73.3 (C_q)	–
9,10	29.2 (CH_3)	1.23 (s, 6H)

[a] Carbon numbering as shown in Figure 3. [b] Chemical shifts δ in ppm and assignment of carbons by ^{13}C-DEPT135 spectroscopy. [c] Chemical shifts δ in ppm, multiplicity m (s = singlet, m = multiplet). Data are in agreement with those reported in literature [4].

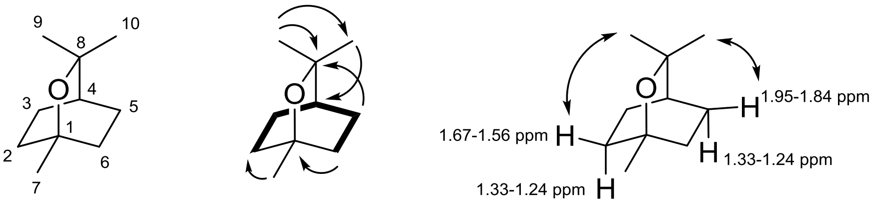

Figure S3: Structure and carbon numbers of 1,8-cineol (**1**). H,H-COSY spin systems are indicated by bold lines, single headed arrows represent HMBC correlations and key NOESY correlations are symbolised by double headed arrows.

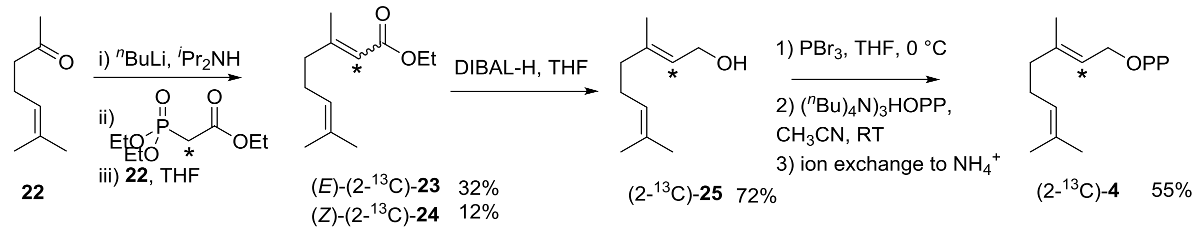

Scheme S2: Synthesis of (2-^{13}C)GPP by phosphorylation of the corresponding (2-^{13}C)geraniol [5]. ^{13}C labelled positions are indicated by asterisks.

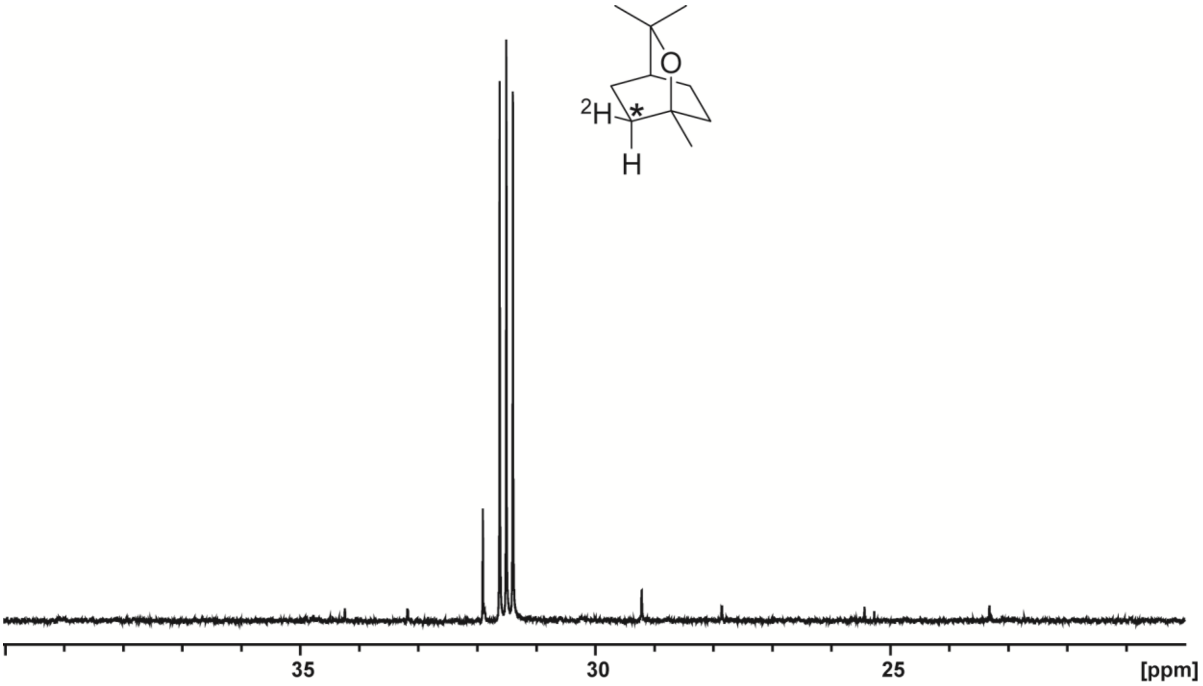

Figure S4: ^{13}C NMR spectrum of (2-^{13}C,2-^{2}H)-**1** arising from incubation of 1,8-cineol synthase with (2-^{13}C)GPP in the presence of ^2H_2O. The ^{13}C-labelled carbon atom is highlighted by an asterisk.

References

[1] a) Edelstein, R. L.; Weller, V. A.; Distefano, M. D.; Tung, J. S. *J. Org. Chem.,* **1998,** *63,* 5298–5299; b) Thulasiram, H. V.; Phan, R. M.; Rivera, S. B.; Poulter, C. D. *J. Org. Chem.,* **2006,** *71,* 1739–1741; c) Keller, R. K.; Thompson, R.; *J. Chromatogr. A,* **1993,** *645,* 161–167.

[2] Hoye, T. R.; Jeffrey, C. S.; Shao, F. *Nat. Protoc.,* **2007,** *2,* 2451–2458.

[3] Juliani, H. R.; Zygadlo, J. A.; Scrivanti, R.; de la Sota, E.; Simon, J. E. *Flavour Fragr. J.,* **2004,** *19,* 541–543.

[4] Bohlmann, F.; Zeisberg, R.; Klein, E. *Org. Magn. Res.* **1975,** *7,* 426–432.

[5] Rabe, P.; Barra, L.; Rinkel, J.; Riclea, R.; Citron, C. A.; Klapschinski, T. A.; Janusko, A.; Dickschat, J. S. *Angew. Chem. Int. Ed.,* **2015,** *54,* 13448–13451.
